# Genome-Guided Characterization of *Ochrobactrum* sp. POC9 Enhancing Sewage Sludge Utilization—Biotechnological Potential and Biosafety Considerations

**DOI:** 10.3390/ijerph15071501

**Published:** 2018-07-16

**Authors:** Krzysztof Poszytek, Joanna Karczewska-Golec, Anna Ciok, Przemyslaw Decewicz, Mikolaj Dziurzynski, Adrian Gorecki, Grazyna Jakusz, Tomasz Krucon, Pola Lomza, Krzysztof Romaniuk, Michal Styczynski, Zhendong Yang, Lukasz Drewniak, Lukasz Dziewit

**Affiliations:** 1 Laboratory of Environmental Pollution Analysis, Faculty of Biology, University of Warsaw, Miecznikowa 1, 02-096 Warsaw, Poland; kposzytek@biol.uw.edu.pl (K.P.); karczewska@biol.uw.edu.pl (J.K.-G.); grazyna.jakusz@biol.uw.edu.pl (G.J.); tkrucon@biol.uw.edu.pl (T.K.); pola.lomza@biol.uw.edu.pl (P.L.); zyang@biol.uw.edu.pl (Z.Y.); ldrewniak@biol.uw.edu.pl (L.D.); 2 Department of Bacterial Genetics, Institute of Microbiology, Faculty of Biology, University of Warsaw, Miecznikowa 1, 02-096 Warsaw, Poland; aciok@biol.uw.edu.pl (A.C.); decewicz@biol.uw.edu.pl (P.D.); mikolaj.dziurzynski@biol.uw.edu.pl (M.D.); agorecki@biol.uw.edu.pl (A.G.); romaniuk@biol.uw.edu.pl (K.R.); mstyczynski@biol.uw.edu.pl (M.S.)

**Keywords:** antibiotic resistance, biosafety, biogas production, *Ochrobactrum* sp. POC9, methane, sewage sludge utilization

## Abstract

Sewage sludge is an abundant source of microorganisms that are metabolically active against numerous contaminants, and thus possibly useful in environmental biotechnologies. However, amongst the sewage sludge isolates, pathogenic bacteria can potentially be found, and such isolates should therefore be carefully tested before their application. A novel bacterial strain, *Ochrobactrum* sp. POC9, was isolated from a sewage sludge sample collected from a wastewater treatment plant. The strain exhibited lipolytic, proteolytic, cellulolytic, and amylolytic activities, which supports its application in biodegradation of complex organic compounds. We demonstrated that bioaugmentation with this strain substantially improved the overall biogas production and methane content during anaerobic digestion of sewage sludge. The POC9 genome content analysis provided a deeper insight into the biotechnological potential of this bacterium and revealed that it is a metalotolerant and a biofilm-producing strain capable of utilizing various toxic compounds. The strain is resistant to rifampicin, chloramphenicol and β-lactams. The corresponding antibiotic resistance genes (including *bla_OCH_* and *cmlA/floR*) were identified in the POC9 genome. Nevertheless, as only few genes in the POC9 genome might be linked to pathogenicity, and none of those genes is a critical virulence factor found in severe pathogens, the strain appears safe for application in environmental biotechnologies.

## 1. Introduction

Natural and anthropogenically-shaped environments are frequently screened for the presence of bacterial strains potentially useful in environmental biotechnologies [1,2,3,4]. Municipal wastewaters and the products of their transformation (including sewage sludge)—as the environments rich in various toxic compounds—are potentially a good source of microorganisms metabolically active against numerous contaminants. Such microorganisms are well adapted to a variety of life-limiting factors, including toxic organic compounds and heavy metals [5,6,7], and thus are desired in technologies dedicated to sewage sludge transformation and utilization. However, pathogenic and antibiotic-resistant strains can also be found amongst bacteria isolated from wastewaters [8,9,10]. Therefore, for biosafety reasons, each isolate should be thoroughly analysed and, ideally, its genome should be explored to track, e.g., antibiotic resistance and virulence genes.

*Ochrobactrum* spp. are aerobic, non-fermenting, Gram-negative bacteria thriving in various environments, including soil, water, plants, and animals [11,12,13,14,15]. Members of the *Ochrobactrum* genus have frequently been isolated from environments persisting under strong anthropogenic pressure and some representatives were defined as animal and human pathogens. For example, *O. anthropi* and *O. intermedium* were recognized as relatively benign opportunistic human pathogens, infecting mostly immunocompromised patients [11,15,16,17,18,19]. However, cases of life-threatening infections, e.g., endocarditis caused by *O. anthropi*, were also reported [20]. *Ochrobactrum* spp. are metabolically versatile and exhibit some unique features, e.g., *O. intermedium* MZV101 is able to produce lipase and biosurfactants at pH 10 and temperature of 60 °C [21]. What is more, *Ochrobactrum* spp. seem to be well adapted for living in contaminated environments, which are rich in xenobiotics, organic pollutants and heavy metals. *Ochrobactrum* strains produce a variety of hydrolyzing enzymes that enable them to utilize even hardly-degradable compounds, e.g., phenols, organophosphorus pesticides and petroleum hydrocarbons [22,23,24,25,26]. Therefore, bacteria of this genus gained attention as promising candidates for use in diverse fields of biotechnology. They have been frequently employed in bioremediation technologies, e.g., in continuously stirred tank bioreactors to treat hydrocarbon-rich industrial wastewaters, or in biopiles and land farming to remove petroleum pollutions [27,28,29,30].

In this study, a novel *Ochrobactrum* strain, *Ochrobactrum* sp. POC9, was isolated from sewage sludge originated from the wastewater treatment plant (WWTP) “Czajka” in Warsaw, Poland. The strain exhibited various enzymatic activities, and significantly enhanced biogas production by improving sewage sludge utilization. The draft genomic sequence of *Ochrobactrum* sp. POC9 was obtained and thoroughly analyzed, which—together with functional analyses—provided insight into the biotechnological potential and the biosafety of the strain.

## 2. Materials and Methods

### 2.1. Isolation of Ochrobactrum sp. POC9, Culture Conditions and Screening of Enzymatic Activities

A raw sewage sludge sample collected from the wastewater treatment plant “Czajka” (Warsaw, Poland) was serially diluted using 0.8% (*w*/*v*) saline solution. Then, 100 μL of each dilution was spread on lysogeny broth (LB) solidified by the addition of 1.5% (*w*/*v*) agar [31]. The plates were incubated at 37 °C for 72 h. A random selection of 100 isolates differing in their colony morphology was subjected to enzymatic activity examination. For testing of the proteolytic activity, Frazier agar (BTL, Lodz, Poland) and nutrient agar medium [31] with skim milk (10% *v*/*v*) were used. For screening of the lipolytic activity, tributyrin agar (Sigma-Aldrich, St. Louis, MO, USA) was used. For screening of the cellulolytic and amylolytic activities, CMC-Red Congo agar [32] and nutrient agar medium [31] supplemented with a specific soluble chromogenic substrate (Megazyme, Bray, Ireland), respectively, were used. In each case, the material from a single colony was transferred onto the specific medium and the plates were incubated at 37 °C for 72 h, during which the visual observation was performed. Formation of clearing zones around the bacterial colonies indicated the hydrolysis of a particular substrate. Each experiment was carried out in triplicate. Based on the results of the performed analyses, the fastest growing strain (*Ochrobactrium* sp. POC9) exhibiting proteolytic, lipolytic, cellulolytic and amylolytic activities was selected for further studies.

### 2.2. Amplification and Sequencing of the 16S rRNA Gene

Genomic DNA was extracted from the bacterial cells using Genomic Mini purification kit (A&A Biotechnology, Gdynia, Poland). The 16S rRNA gene fragment was amplified by PCR with universal primers 27f and 1492r [33]. The amplified 16S rDNA fragment was used as a template for DNA sequencing with ABI3730xl DNA Analyzer (Applied Biosystems, Thermo Fisher Scientific, Foster City, CA, USA).

### 2.3. Draft Genome Sequencing

Genomic DNA of the POC9 strain was isolated using the CTAB/Lysozyme method [31]. An Illumina TruSeq library was constructed following the manufacturer’s instructions. The genomic libraries were sequenced on Illumina MiSeq instrument (using the v3 chemistry kit) (Illumina, San Diego, CA, USA) in the DNA Sequencing and Oligonucleotide Synthesis Laboratory (oligo.pl) at the Institute of Biochemistry and Biophysics, Polish Academy of Sciences, Warsaw. The reads trimmed with CutAdapt v 1.9.1 [34] were further assembled using Newbler De Novo Assembler v3.0 (Roche, Basel, Switzerland).

### 2.4. Bioinformatic Analyses

The POC9 genome was automatically annotated using RAST [35] on PATRIC 3.5.11 [36] web service. Similarity searches were performed using BLAST programs [37] and Pfam database [38]. Metabolic features were identified applying KEGG database [39]. The COG numbers were assigned to each gene by local RPS-BLAST search against the COG database (last modified 22 January 2015) with 1e-5 e-value threshold by considering only the best BLAST hits [40]. Putative rRNA and tRNA sequences were identified using the Rfam [41], tRNAScan-SE [42] and ARAGORN programs [43]. To identify genetic determinants responsible for the heavy metal resistance phenotype, the genome was screened using the BacMet: antibacterial biocide and metal resistance genes database [44]. To identify antibiotic resistance genes, the Resistance Gene Identifier (RGI; Comprehensive Antibiotic Resistance Database) software was used [45]. Potential virulence factors were identified using VFDB–Virulence Factors Database [46].

### 2.5. Analytical Methods

Dry organic matter (VS) analysis was performed according to standard methods described by the American Public Health Association (APHA, 1998) [47]. The volatile fatty acids (VFAs) concentration and soluble chemical oxygen demand (sCOD) were determined using Nanocolor^®^ kits (Machery-Nagel GmbH, Düren, Germany). The volume of the produced biogas was monitored using Milligascounter MGC-1 (Ritter, Bochum, Germany). Methane content was analyzed with a gas analyzer GA5000 (Geotech, Leamington Spa, UK).

### 2.6. Simulation of the Anaerobic Digestion Process

The effect of bioaugmentation of sewage sludge anaerobic digestion with *Ochrobactrum* sp. POC9 was investigated in laboratory-scale anaerobic batch experiments, which were performed in 1-L glass bottles GL 45 (SCHOTT Poland, Warsaw, Poland) connected with Dreschel-type scrubbers. To each reactor, a 1-L Tedlar gas bag (Sigma-Aldrich, St. Louis, MO, USA) was attached to collect biogas. Each bioreactor was filled with (i) the liquid phase from a separated fermentation chamber from the wastewater treatment plant “Krym” (Wolomin, Poland) [11 g dry organic matter per l (11 gvs∙L^−1^)], containing a methanogenic consortium inoculum and (ii) sewage sludge from the same WWTP [11 g dry organic matter per l (11 gvs L^−1^)]. Then, bioreactors were supplemented with 4 mL of the bacterial (*Ochrobactrum* sp. POC9) suspension (approx. 10^7^ cells∙mL^−1^). The negative controls for the experiment were cultures containing only methanogenic consortium and sewage sludge from the WWTP “Krym” without the addition of *Ochrobactrum* sp. POC9. At the beginning of the experiment, the total dry organic matter content in all bioreactors was 22 gvs∙L^−1^, soluble chemical oxygen demand (sCOD) was 1.97 g L^−1^ and VFAs concentration was 5.03 g∙L^−1^. Anaerobic batch assays were run at 37 °C for 30 days without refeeding. Analyses were carried out at the beginning of the experiment and after 3, 7, 14, 21, and 30 days. The experiment was performed in triplicate.

### 2.7. Antibiotic Susceptibility Testing

To determine the antimicrobial susceptibility patterns of *Ochrobactrum* sp. POC9, MICs of 10 antimicrobial agents were assessed using Etest™ (Liofilchem, Roseto degli Abruzzi, Italy). The analysis was conducted according to European Committee on Antimicrobial Susceptibility Testing (EUCAST) recommendations [48]. The following antibiotics (selected based on bioinformatic analyses that identified putative antibiotic resistance genes) were used: aminoglycosides–gentamicin (CN; concentration of antibiotic: 0.064–1024 µg mL^−1^), β-lactams (penicillin derivatives)–ampicillin (AMP; 0.016–256 µg∙mL^−1^), β-lactams (cephalosporins)–cefixime (CFM; 0.016–256 µg∙mL^−1^), β-lactams (cephalosporins)–cefotaxime (CTX; 0.016–256 µg∙mL^−1^), β-lactams (cephalosporins)–ceftriaxone (CRO; 0.016–256 µg mL^−1^), fluroquinolones–ciprofloxacin (CIP; 0.002–32 µg∙mL^−1^), fluroquinolones–moxifloxacin (MXF; 0.002–32 µg∙mL^−1^), phenicols–chloramphenicol (C; 0.016–256 µg∙mL^−1^), ryfamicins–rifampicin (RD; 0.016–256 µg∙mL^−1^), tetracyclines–tetracycline (TE; 0.016–256 µg∙mL^−1^). The susceptibility testing was performed at 37 °C for 20 h. After incubation, plates were photographed and MICs were defined. Antimicrobial susceptibility data were interpreted according to the EUCAST breakpoint table (version 8.0) [48].

### 2.8. Heavy Metal Resistance Testing

Analytical grade salts (NaAsO_2_, Na_2_HAsO_4_ × 7H_2_O, 3CdSO_4_ × 8H_2_O, CoSO_4_ × 7H_2_O, K_2_CrO_4_, CuSO_4_, NiSO_4_ × 7H_2_O, ZnSO_4_ × 7 H_2_O) (Sigma-Aldrich) were used in a resistance assay performed in 96-well plates, as described previously [49]. Triplicate cultures of the tested strain were challenged with a range of concentrations of those heavy metal salts. If the strain grew in the presence of the following heavy metal ion concentrations, it was considered resistant: (i) 10 mM As (V), (ii) 1 mM Cd^2+^, Co^2+^, CrO4^2−^, Cu^2+^, Ni^2+^, Zn^2+^, (iii) 2.5 mM As (III) [50,51,52,53].

### 2.9. Adherence of Bacteria to Artificial Surface (Biofilm Formation) Testing

The modified crystal violet staining method was used [54]. Bacteria were cultivated overnight in LB medium at 37 °C and then diluted to obtain OD (optical density) = 0.1 at 600 nm (OD_600_; CFU ≈ 1.3 × 10^8^) in the same medium. In the next step, 200 μL of three biological replicates were transferred into 24 sterile 96-well plates. Bacteria were incubated at 37 °C for 24 h, 48 h, and 72 h. The OD_600_ of cultures was then measured using Sunrise^TM^ plate reader (Tecan, Männedorf, Switzerland) with Magellan software (Tecan). In the next step, the medium with pelagic cells (i.e., cells that did not adhere to artificial surface, and thus were not a part of a formed biofilm or alternatively were released from a formed biofilm) from each well was removed, and the wells were rinsed with saline solution and dried at 37 °C for about 15 min. The adhered (biofilm-forming) bacteria were stained with crystal violet (200 μL/well) for 10 min at room temperature. Then, an excess dye was removed, wells were rinsed with saline solution, and dried again at 37 °C. Dried and stained biofilm was then dissolved with 98% ethanol (200 μL/well). The OD_570_ measurement of the obtained suspension was carried out using a Sunrise^TM^ instrument equipped with Magellan software. The significance of statistical results was determined by the Student *t*-test [55].

### 2.10. Nucleotide Sequence Accession Number

The whole-genome shotgun project of *Ochrobactrum* sp. POC9 has been deposited in the NCBI GenBank (https://www.ncbi.nlm.nih.gov/genbank) database under the accession number SAMN09237519.

## 3. Results and Discussion

### 3.1. Isolation and Identification of Ochrobactrum sp. POC9

*Ochrobactrum* sp. POC9 was isolated from raw sewage sludge collected from the “Czajka” wastewater treatment plant (Warsaw, Poland) in 2014. An initial screening for culturable bacteria was aimed at isolating strains capable of utilizing a broad spectrum of organic compounds. In such preliminary tests, the POC9 strain exhibited unequivocal lipolytic, proteolytic, cellulolytic, and amylolytic activities. For identification of this bacterium, its 16S rRNA gene was amplified and sequenced. The obtained sequence was compared with the 16S rDNA sequences gathered in the NCBI database, and the POC9 strain was classified into the *Ochrobactrum* genus.

**Figure 1:**
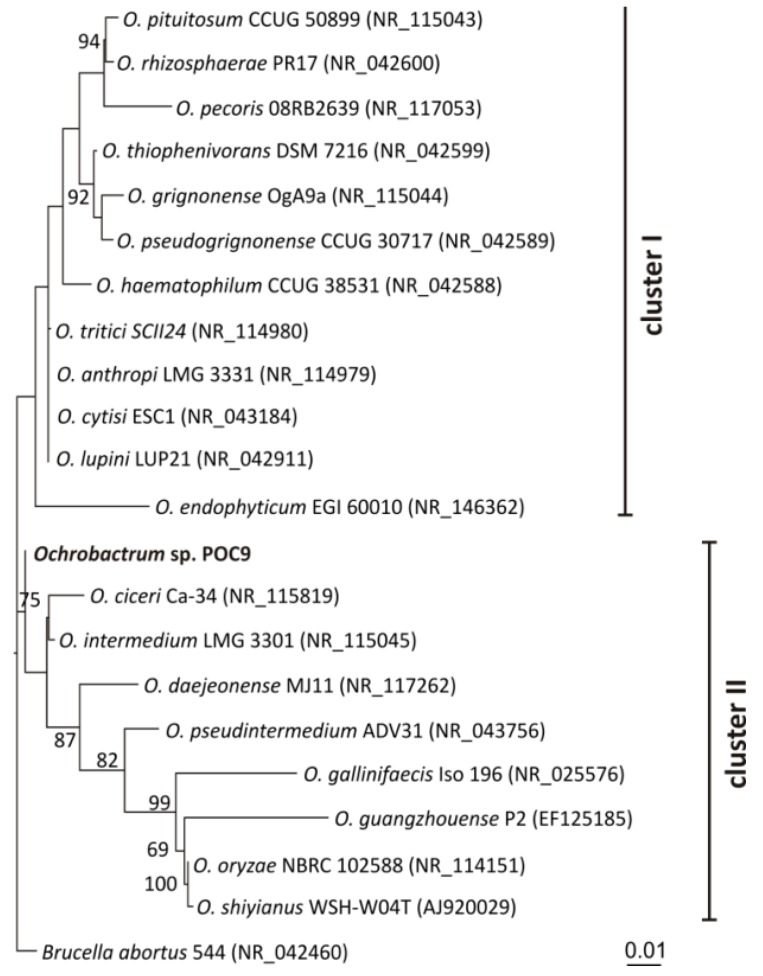
Phylogenetic tree for 16S rDNA sequences of *Ochrobactrum* spp. The tree was constructed by applying the Maximum Likelihood method based on the Tamura-Nei model. Statistical support for the internal nodes was determined by 1000 bootstrap replicates and values of ≥50% are shown. Initial tree(s) for the heuristic search were obtained automatically by applying Neighbor-Join and BioNJ algorithms to a matrix of pairwise distances estimated using the Maximum Composite Likelihood (MCL) approach. A discrete Gamma distribution was used to model evolutionary rate differences among sites (5 categories (+G, parameter = 0.6173)). The tree is drawn to scale, with branch lengths measured in the number of substitutions per site. The analysis involved 22 nucleotide sequences with 16S rDNA sequence of *Brucella abortus* 544 used as an outlier. All positions containing gaps and missing data were eliminated. There were a total of 1353 positions in the final dataset. GenBank accession numbers of the 16S rDNA sequences used for the phylogenetic analysis are given in parentheses. The 16S rDNA of the POC9 strain, analyzed in this study, is in bold text.

A phylogenetic analysis based on 16S rDNA sequences of the POC9 strain and 20 other reference *Ochrobactrum* species was then performed. Analysis of the phylogenetic tree topology revealed the presence of two separate clusters. The first one gathered 12 species with the *O. endophyticum* EGI 60010 as an outlier. The second cluster grouped nine representatives of *Ochrobactrum* spp. with *Ochrobactrum* sp. POC9 as an outlier. Based on 16S rDNA sequence analysis results we may speculate that *Ochrobactrum* sp. POC9 is most closely related to *O. intermedium* and *O. ciceri* (Figure 1).

### 3.2. Bioaugmentation of Sewage Sludge Anaerobic Digestion

Bioaugmenation is a method that relies on adding specific microorganisms or microbial consortia to biological systems to enhance a desired activity [56]. This technology can be also applied in anaerobic digestions, e.g., to increase biogas (methane) production. Examples of various bioaugmentation procedures applied in anaerobic digestion of wastes are presented in Table 1.

Lipids, proteins and polysaccharides in sewage sludge are a potent source of energy and substrates for biogas production [64]. However, such use of sewage sludge is limited due to its heterogeneity, complex structure, and the presence of toxic compounds, e.g., heavy metals [65]. The initial breakdown of sewage sludge components can be substantially improved by the addition of bacteria capable of hydrolyzing complex compounds [66].

The POC9 strain exhibited diverse enzymatic (lipolytic, proteolytic, cellulolytic and amylolytic) activities under laboratory conditions. To determine whether the strain is able to break down complex residues present in sewage sludge, and thus whether it could be used for bioaugmentation of anaerobic digestion of sewage sludge, batch experiments were performed. The bioaugmentation with *Ochrobactrum* sp. POC9 was carried out once, at the beginning of the experiment. During a 30-day simulation of anaerobic digestion of sewage sludge, the daily biogas production yield and methane content in biogas were measured.

The cumulative biogas production in cultures supplemented with the POC9 strain increased by 22.06% compared to the control (non-bioaugmented) cultures. The cumulative biogas production for anaerobic digestion of sewage sludge with the addition of *Ochrobactrum* sp. POC9 was 294.58 ± 44.98 dm^3^/kg of VS, while in the control experiment it was 229.58 ± 13.92 dm^3^/kg (Table 2). Interestingly, the analysis of the daily biogas production revealed that the increased biogas production occurred mostly during the first eight days of culturing (Figure S1).

The methane content during anaerobic digestion of sewage sludge after bioaugmentation with *Ochrobactrum* sp. POC9 increased from 46.00% in the third day of culturing to 58.87% after 30 days of culturing, and was highest on the seventh day—66.48% (Table 2). At the same time, methane content in biogas in the control variant increased from 43.41% to 49.18%, and was highest also on the seventh day, when it reached 61.34% (Table 2).

The results showed that the bioaugmentation with the POC9 strain improved the overall biogas production and methane content during anaerobic utilization of sewage sludge. This might be a consequence of improved hydrolysis occurring at the first stage of an anaerobic decomposition process. During this step, the combined lipolytic, proteolytic, cellulolytic and amylolytic activities of the POC9 strain probably enabled specific “pretreatment” of the raw sewage sludge, leading to increased production of easily utilizable, simple compounds (e.g., simple sugars, fatty acids, amino acids, etc.) that may be readily used by indigenous microorganisms (i.e., acetogenic bacteria and metahnogenic arachaea) for biogas production.

### 3.3. Genome-Based Insight into the Metabolic Potential of the POC9 Strain

Sequencing of the *Ochrobactrum* sp. POC9 genome on the Illumina MiSeq platform generated 1,275,451 paired-reads and 76,6385,632 nucleotides. As the result of the assembly, 298 contigs of a total length of 4,976,112 bp were obtained. The genome sequence was automatically annotated using RAST on PATRIC 3.5.11 web service and its general features are presented in Table 3. Based on the results of the manual inspection of the genome assembly and annotation, three potential plasmid contigs: contig00029 (35,460 bp; QGST01000029.1), contig00037 (31,160 bp; QGST01000037.1), and contig00057 (5064 bp; QGST01000057.1) were identified. In these contigs, complete *repABC*-type replication-partitioning modules, typical of large replicons of *Alphaproteobacteria*, were found [67].

**Figure 2:**
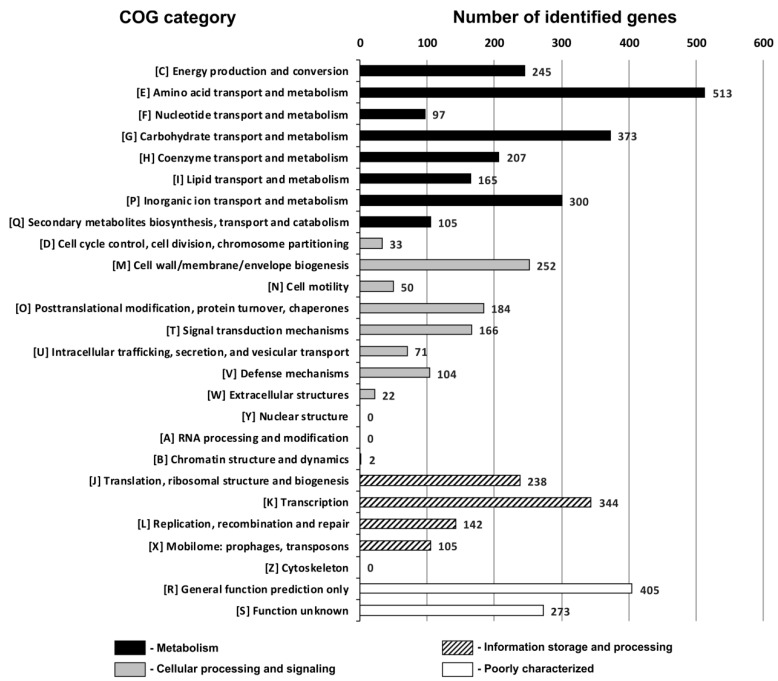
Number of genes classified into particular COG functional categories.

Genes identified within the POC9 genome were blasted against the COG database [40]. COG numbers were assigned to 3894 genes, considering only the best RPSBLAST hit with the e-value threshold of 1e-5. The genes were classified into appropriate COG categories (Figure 2).

This analysis revealed that 2005 (51.5%) genes with assigned COG numbers were associated with the cellular metabolism, and the most numerous fractions of the classified genes were: E (grouping proteins associated with amino acid transport and metabolism)—13.2%, G (carbohydrate transport and metabolism)—9.6%, and P (inorganic ion transport and metabolism)—7.7%. These observations suggested that the POC9 strain possesses complex metabolic networks and is able to utilize or transform a broad spectrum of organic and inorganic compounds.

To gain a deeper insight into the POC9 metabolic potential, its draft genome was subjected to an analysis with KEGG Automatic Annotation System (KAAS), which allowed for reconstruction of its metabolic pathways. Apart from the basic metabolic pathways, such as glycolysis and the Krebs cycle, the KEGG-mapping revealed the presence of genes encoding both the enzymes enabling utilization of complex and toxic compounds and other features important in biotechnological applications, and especially advantageous in sewage sludge utilization. *Ochrobactrum* sp. POC9 possesses complete gene repertoires for the following pathways: (i) phenol degradation through benzoate utilization [23], (ii) propanoate utilization [68], (iii) denitrification [69], and (iv) assimilatory sulfate reduction [70] (Figure S2). Given the potential application of *Ochrobactrum* sp. POC9 for enhanced biogas production, it is also important to emphasize its possible contribution to CO_2_ availability for methanogenesis. The POC9 strain can potentially produce CO_2_ in the citric acid cycle and also through decarboxylation of formate, formaldehyde, and *N*-formyl derivatives [71,72] (Figure S2).

### 3.4. Heavy Metal Metabolism

We demonstrated that *Ochrobactrum* sp. POC9 enhances sewage sludge utilization. However, sewage sludge usually contains various toxic contaminants, including heavy metals and metalloids (e.g., As, Cd, Co, Cr, Cu, Fe, Hg, Ni, Pb and Zn) [73,74,75]. The presence of such toxic compounds may decrease the overall efficiency of the utilization treatment due to their bactericidal effect on the exogenous microorganisms used in the treatment. Hence, bacterial strains used in bioaugmentation during sewage sludge utilization should tolerate heavy metals at the highest possible concentrations [76].

Therefore, in the next step, the POC9 genome was screened for the presence of genes putatively involved in heavy metal ions transformation and resistance. We found 10 such genetic modules, encoding: (i–vi) six heavy metal translocating P-type ATPases ZntA (COG2217), which may potentially confer resistance to various divalent ions, i.e., Cd(II), Co(II), Cu(II), Ni(II), Pb(II), and Zn(II) [77,78,79]; (vii) chromate transport protein ChrA (COG2059), which actively transports chromate ions across cell membrane [80]; (viii–ix) two divalent metal ion transporters of the cation diffusion facilitator (CDF) family, i.e., FieF (COG0053), which usually function as Fe(II) efflux system but may also confer resistance to Cd(II), Co(II), Ni(II), and Zn(II), as well as CzcD (COG1230), which protects the cell from Cd(II), Co(II), and Zn(II) [81,82]; and (x) arsenic resistance module (ARS), which may be involved in resistance to arsenate and arsenite (Table 4). The ARS module encodes (i) arsenite efflux pump ArsB (COG0798) transporting As(III) ions out of the cell, (ii) arsenate reductase ArsC (COG1393), which reduces As(V) to As(III), and (iii) ArsH, the function of which is not completely understood, but it was hypothesized that the protein may play a regulatory role [83].

We tested whether the predicted genes and gene clusters identified in the POC9 genome are able to confer resistance to heavy metals. The effect of As(III), As(V), Cd(II), Co(II), Cr(VI), Cu(II), Ni(II), and Zn(II) ions on *Ochrobactrum* sp. POC9 growth was examined. These metals were selected based on the predicted metal specificity of the putative resistance genes identified within the POC9 genome (Table 4). The MIC values were determined to establish the levels of metal resistance of the strain. The obtained results are as follows: As(III)—MIC value of 3 mM, As(V)—1200 mM, Cd(II)—1 mM, Co(II)—1 mM, Cr(VI)—3 mM, Cu(II)—5 mM, Ni(II)—2 mM, and Zn(II)—3 mM.

*Ochrobactrum* sp. POC9 exhibited an extremely high-level of As(V) resistance and a moderate level of As(III) resistance. The observed resistance to arsenic compounds is probably mediated by the presence of the ARS module enabling reduction of arsenate to arsenite and its further extrusion from the cell. The POC9 strain showed also a moderate level of resistance to Cr(VI), Cu(II), Ni(II), and Zn(II) ions, which may be linked to the presence of the above mentioned transporters of broad-range specificities, i.e., ChrA, CzcD, FieF, and ZntA.

The results indicated that the POC9 strain shows at least moderate resistance to six heavy metal ions that are frequently present in sewage sludge. This makes it a robust candidate for application in biotreatment of metal-contaminated waste, as the strain is well equipped to withstand the toxic effect of heavy metals in these environments.

### 3.5. Antibiotic Resistance Genes and Virulence Factors

Wastewater treatment plants are considered a point source of emerging pollutants, including antibiotics, antibiotic resistant and/or pathogenic bacteria as well as antibiotic resistance and virulence genes [84]. Therefore, with an aim to apply *Ochrobactrum* sp. POC9—a wastewater isolate–in biotechnology, and taking into account biosafety considerations, genome of this strain was analyzed for the presence of antibiotic resistance and virulence genes.

To determine the antimicrobial resistance profile of *Ochrobactrum* sp. POC9, bioinformatic analyses and MIC characterization using Etests were performed. It total, five antibiotic resistance genes (*bla_OCH_*, *qacH*, *cmlA/floR*, *acc (6’)*, and *tetG*) and two clusters of genes encoding a multidrug efflux system (*acrAB-TolC*) potentially conferring resistance to various types of β-lactams, aminoglycosides, fluoroquinolones, tetracyclines, phenicols and ryfamicins were identified within the POC9 genome (Table 5).

Results of the analysis with Etests indicated that *Ochrobactrum* sp. POC9 is resistant to various β-lactams (including ampicillin, cefexime, cefotaxime, and ceftriaxone), rifampicin and chloramphenicol (Table 5). Broad spectrum resistance to various classes of β-lactams was previously observed in other (also non-pathogenic) *Ochrobactrum* strains [85]. Resistance phenotype in those strains was linked to the presence of a class C β-lactamase gene (*bla_OCH_*) and to an upstream gene encoding a LysR family regulator [85]. Within the POC9 genome, the *bla_OCH_* gene and an upstream regulatory *gcvA* gene were also found. Interestingly, protein products of these genes in POC9 exhibited 98% sequence identity with class C beta-lactamase of *Ochrobactrum tritci* (GenBank acc. no. SME85995) and transcriptional regulator GcvA of *Ochrobactrum* anthropi (GeneBank acc. no. WP_061347328). It seems that the presence of highly conserved *bla_OCH_* gene is common in *Ochrobactrum* genomes. Furthermore, the POC9 strain was also resistant to chloramphenicol, which well reflects the presence of the *cmlA/floR* gene in its genome [86].

The Etest results revealed that the POC9 strain was susceptible to ciprofloxacin, moxifloxacin, gentamicin and tetracycline, which suggests that *qacH*, *acc (6’)* and *tetG* were inactive under the tested laboratory conditions (Table 5). Further analyses with a broader range of antibiotics belonging to fluoroquinolones, aminoglycosides and tetracyclines should be carried out to test whether these genes are specific to other antibiotics of these groups.

The results of the susceptibility testing did not enable us to unequivocally determine whether the above mentioned *acrAB*-*TolC* modules encoding resistance-nodulation-cell division (RND) type multidrug efflux systems are active or not, as their predicted activities partially overlap with the specificities of proteins encoded by *bla_OCH_* and *cmlA/floR* genes [85,86,87] (Table 5).

The POC9 genome was also screened for the presence of putative virulence genes. Only a few genetic modules that may potentially be linked to pathogenicity were found. These were: (i–iii) Sec-SRP (the general secretion route), Tat (Twin-arginine translocation pathway), Vir-like type IV secretion systems (T4SS) [88], and (iv–v) genes responsible for the synthesis of flagella and sigma-fimbriae subsystem (sF-Chap, sF-UshP, sF-Adh), which may be responsible for biofilm formation and adherence of cells to various surfaces [89]. Within the genome of *Ochrobactrum* sp. POC9, we also identified several genes encoding proteins responsible for the synthesis of lipopolysaccharides (LpxA, LpxB, LpxC, LpxD, LpxH, LpxK, LpxL and KdtA) as well as ABC-type systems (*rfb* and *lpt*) responsible for the transport of lipopolysaccharides [90,91,92]. Whereas these genes may be somehow linked to the POC9 strain pathogenicity, they are common among *Alphaproteobacteria*, including non-pathogenic strains [93].

The presence of genes involved in the synthesis of lipopolysaccharides constituting biofilm matrix as well as several genes that may indirectly influence biofilm formation (e.g., sigma-fimbriae subsystem) inspired us to analyze biofilm formation ability in the POC9 strain. Whereas bacterial biofilms are usually linked to the virulence of clinical strains, they also play a major ecological role in maintaining complex multispecies ecosystems in diverse environments [94]. Moreover, biofilms are also beneficial in bioremediation, e.g., they are crucial for the proper functioning of the attached growth systems in WWTPs [95]. The crystal violet staining method was applied to determine the adherence potential of *Ochrobactrum* sp. POC9. The results showed that the POC9 strain is able to adhere to surface after 24 h of incubation without shaking and then maintains the biofilm up to 48 h of incubation. Interestingly, after 72 h, the biofilm began to disperse, resulting in a significant (*p* < 0.005) reduction of the adhered biomass (Figure S3). This observation suggested that the POC9 strain is able to form biofilm or to at least initiate its formation by adhesion to artificial surfaces. However, stable maintenance of the biofilm may require specific (and not yet recognized) environmental conditions or cooperation with other microorganisms.

## 4. Conclusions

In this study, a novel bacterial strain, *Ochrobactrum* sp. POC9, was isolated from sewage sludge. The strain exhibited diverse enzymatic (lipolytic, proteolytic, cellulolytic and amylolytic) activities, which suggested that it might be beneficial in the biodegradation of complex organic compounds. Therefore, the strain was tested for its ability to enhance the process of sewage sludge utilization, and was found to substantially improve the overall biogas yield and methane content during anaerobic digestion of sewage sludge. This may be a consequence of enhanced hydrolysis occurring at the first stage of the process. The obtained results suggest that *Ochrobactrum* sp. POC9 carries out specific “pretreatment” of the raw sewage sludge, which leads to increased production of easily utilizable simple organic compounds for other microorganisms (i.e., acetogenic bacteria and methanogenic arachaea) involved in the biogas production.

The analysis of the POC9 genome content offered a deeper insight into the biotechnological potential of this bacterium and revealed its denitrifying, biofilm forming, and toxic compound (e.g., phenol) utilization abilities. Genomic investigations combined with physiological analyses indicated also that *Ochrobactrum* sp. POC9 is a metalotolerant bacterium, and carries several heavy metal resistance genes in its genome. This is a beneficial feature, as heavy metals are common in wastes, and may generate bactericidal effect. We also demonstrated that the POC9 strain is resistant to various β-lactams (including ampicillin, cefexime, cefotaxime, and ceftriaxone), rifampicin and chloramphenicol, which well correlates with the presence of several antibiotic resistance genes, including *bla_OCH_* and *cmlA/floR*, in the *Ochrobactrum* POC9 genome. Nevertheless, as only few genes in the POC9 genome were recognized as potentially linked to pathogenicity, and none of these genes is a critical virulence factor found in severe pathogens, the strain appears safe for environmental biotechnology applications.

## 5. Patents

Polish patent No. PL413998. Drewniak L., Poszytek K., Dziewit L., Sklodowska A. 2018. Consortium of microorganisms capable of hydrolysis of the proteins and lipids in the sewage sludge and/or contaminated soil, the formulation comprising them, the application of the consortium and method of hydrolysis of proteins, lipids and hardly degradable compounds in sewage sludge and/or organic compounds in soils.

## Figures and Tables

**Table 1:** Examples of various bioaugmentation procedures applied in anaerobic digestion of wastes.

Strain/ Microbial Consortium	Scale	Substrate for Anaerobic Digestion	Effect	Reference
*Caldicellulosiruptor saccharolyticus,* *Enterobacter cloacae*	Laboratory	Waste water sludge, pig manure slurry and dried plant biomass from Jerusalem artichoke	Increased biogas production of up to 160–170%	[57]
*Caldicellulosiruptor lactoaceticus* 6A, *Dictyoglomus* sp. B4a	Laboratory (batch experiments and CSTR bioreactor)	Cattle manure	Increased methane yield of up to 93%	[58]
Hemicellulolytic consortium immobilized on activated zeolite	Laboratory (batch experiments and CSTR bioreactor)	Xylan from birch wood	Increased methane yield of up to 5%	[59]
*Clostridium thermocellum*, *Melioribacter roseus*	Laboratory scale (batch experiments and CSTR bioreactor)	Wheat straw	Increased methane yield of up to 34%	[60]
*Ruminococcus flavefaciens* 007C, *Pseudobutyrivibrio xylanivorans* Mz5T, *Fibrobacter succinogenes* S85 and *Clostridium cellulovorans*	Laboratory	Brewery spent grain	Increased biogas production of up to 5–18%	[61]
*Methanoculleus bourgensis* MS2	Laboratory	Ammonia-rich substrates (mixed pig and chicken manure, slaughterhouse residues, and food industry waste)	Increased methane yield of up to 34%	[62]
Microbial consortium with high cellulolytic activity (MCHCA)	Laboratory (two-stage anaerobic digestion)	Maize silage (lignocellulose biomass)	Increased biogas production of up to 38%, increased methane yield of up to 64%	[63]

**Table 2:** Cumulative biogas production and methane content in biogas of control and POC9-supplemented variants of the experiment simulating anaerobic digestion of sewage sludge.

Parameter (unit)	Control			Culture with the POC9 Strain		
	3 days	7 days	30 days	3 days	7 days	30 days
Cumulative biogas production (L/kgvs)	229.58 ± 13.92			294.58 ± 44.98		
CH_4_ content (%)	43.41	61.34	49.18	46.00	66.48	58.87

**Table 3:** General features of the *Ochrobactrum* sp. POC9 draft genome.

Genomic feature	Calculation
Number of contigs	298
Estimated genome size (bp)	4,976,112
GC content (%)	55.68%
Coding density (%)	89.07%
Number of genes	5217
Number of tRNA genes	66
Number of 16S-23S-5S rRNA clusters	3

**Table 4:** Heavy metal resistance genes identified within the *Ochrobactrum* sp. POC9 draft genome.

Protein Name	Localization within the POC9 Draft Genome Sequence (GenBank acc. no.)	Predicted Protein Function	Homologous Protein Based on Best BLASTP Hit (GenBank acc. no.)
*arsB*	contig00008 (QGST01000008.1) coordinates: 148,637-147,576	Export of As(III) ions	arsenic transporter of *O. anthropi* FRAF13 (KXO76567)
*arsC*	contig00008 (QGST01000008.1) coordinates: 147,579-147,169	Reduction of As(V) to As(III)	arsenate reductase of *O. intermedium* LMG 3301 (EEQ95705)
*arsH*	contig00008 (QGST01000008.1) coordinates: 147,172-146,459	Unknown function, probably regulatory protein	arsenical resistance protein ArsH of *Ochrobactrum* sp. 30A/1000/2015 (PJT26941)
*chrA*	contig00005 (QGST01000005.1) coordinates: 67,497-66,199	Export of Cr(VI)	chromate transporter of *Ochrobactrum* sp. EGD-AQ16 (ERI13917)
*czcD*	contig00031 (QGST01000031.1) coordinates: 6786-7745	Export of Cd(II), Co(II), and Zn(II)	cation transporter of *Ochrobactrum* sp. MYb71 (PQZ25943)
*fieF*	contig00005 (QGST01000005.1) coordinates: 103,790-102,813	Export of Cd(II), Co(II), Fe(II), Ni(II), and Zn(II)	cadmium transporter of *O. anthropi* FRAF13 (KXO76051)
*zntA*	contig00025 (QGST01000025.1) coordinates: 28,104-26,260	Export of Cd(II), Co(II), Cu(II), Ni(II), Pb(II), and Zn(II)	haloacid dehalogenase of *O. anthropi* FRAF13 (KXO73917)
*zntA*	contig00032 (QGST01000032.1) coordinates: 21,766-24,129	Export of Cd(II), Co(II), Cu(II), Ni(II), Pb(II), and Zn(II)	lead, cadmium, zinc and mercury transporting ATPase; copper-translocating P-type ATPase of *O. haematophilum* FI11154 (SPL62610)
*zntA*	contig00034 (QGST01000034.1) coordinates: 5822-7666	Export of Cd(II), Co(II), Cu(II), Ni(II), Pb(II), and Zn(II)	cadmium-translocating P-type ATPase of *O. rhizosphaerae* PR17 (OYR19288)
*zntA*	contig00065 (QGST01000065.1) coordinates: 984-2825	Export of Cd(II), Co(II), Cu(II), Ni(II), Pb(II), and Zn(II)	heavy metal translocating P-type ATPase of *O. anthropi* ATCC 49188 (ABS17306)
*zntA*	contig00001 (QGST01000001.1) coordinates: 97,090-99,573	Export of Cd(II), Co(II), Cu(II), Ni(II), Pb(II), and Zn(II)	ATPase of *O. anthropi* FRAF13 (KXO79927)
*zntA*	contig00004 (QGST01000004.1) coordinates: 22,193-19,692	Export of Cd(II), Co(II), Cu(II), Ni(II), Pb(II), and Zn(II)	copper-translocating P-type ATPase of *O. lupini* LUP21 (OYR29555)

**Table 5:** Antibiotic resistance genes identified within the *Ochrobactrum* sp. POC9 draft genome and the resistance profile of the strain.

Gene/Gene Cluster Name	Localization within the POC9 Draft Genome Sequence (GenBank acc. no.)	Protein	Best BLAST Hits: [% Identity] Organism (GeneBank acc. no.)	Predicted Antimicrobial Resistance Profile	Tested Antibiotics	Profile
*gcvA-* *bla_OCH_*	contig00009 (QGST01000009.1) coordinates: 164,062–165,431	class C beta-lactamase	[98%] *Ochrobactrum tritici* C8846-N36 (SME85995)	penams, penems, cephalosporins, cephamycins, monobactams	AMP CFM CTX CRO	R R R R
		transcriptional regulator GcvA	[98%] *Ochrobactrum anthropi* (WP_061347328)			
*acrAB-* *TolC*	contig00001 (QGST01000001.1) coordinates: 191,438-196,605	transcriptional regulator TetR	[93%] *Ochrobactrum oryzae* (WP_104756164)	tetracyclines, cephalosporins penams, phenicols, ryfamycins, fluoroquinolones	AMP CIP CFM CTX CRO TE MXF RIF	R S R R R S S R
		efflux RND transporter periplasmic adaptor subunit	[98%] *Ochrobactrum anthropi* (WP_061344971)			
		efflux RND transporter permease subunit	[99%] *Ochrobactrum anthropi* (WP_061344972)			
*acrAB-* *TolC*	contig00010 (QGST01000010.1) coordinates: 49,666–54,163	efflux RND transporter periplasmic adaptor unit	[98%] *Ochrobactrum* sp. (WP_024900215)			
		efflux RND transporter permease subunit	[99%] *Ochrobactrum oryzae* (WP_104755654)			
*qacH*	contig00007 (QGST01000007.1) coordinates: 192,104–191,772	efflux SMR transporter	[100%] *Ochrobactrum* sp. (WP_010661279)	fluoroquinolones	CIP MXF	S S
*cmlA/floR*	contig00016 (QGST01000016.1) coordinates: 56,907–55,714	CmlA/floR chloramphenicol efflux MFS transporter	[94%] *Ochrobactrum anthropi* (WP_061345584)	chloramphenicol	C	R
*acc (6’)*	contig00016 (QGST01000016.1) coordinates: 92,931–92,485	aminoglycoside 6’-acetyl-transferase	[93%] *Ochrobactrum anthropi* FRAF13(KXO77791)	aminoglycosides	CN	S
*tetG-* *tetR*	contig00014 (QGST01000014.1) coordinates: 7148–9063	Tet(A/B/C) family MFS transporter	[87%] *Ochrobactrum oryzae* (WP_104755825)	tetracyclines	TE	S
		transcriptional regulator tetR	[89%] *Ochrobactrum oryzae* (WP_104755986)			

Abbreviations: AMP—ampicillin; C—chloramphenicol; CN—gentamicin; CFM—cefixime; CTX—cefotaxime, CRO—ceftriaxone; CIP—ciprofloxacin; TE—tetracycline; MXF—moxifloxacin; RIF—rifampicin; R—resistant; S—susceptible.

## Supplementary Materials

The following are available online at http://www.mdpi.com/1660-4601/15/7/1501/s1, Figure S1: Daily biogas production during anaerobic digestion of sewage sludge. Figure S2: Metabolic pathways of a putative biotechnological value recognized within the POC9 draft genome. Figure S3: Adherence of *Ochrobactrum* sp. POC9 to an artificial (plastic) surface after three time intervals (24 h, 48 h and 72 h).
