# Supplementary material for: Genome-Guided Characterization of Ochrobactrum sp. POC9 Enhancing Sewage Sludge Utilization—Biotechnological Potential and Biosafety Considerations
Source: Int J Environ Res Public Health. 2018 Jul 16;15(7):1501. doi: 10.3390/ijerph15071501 (PMC6069005; doi:10.3390/ijerph15071501)
Supplement: Supplementary file 1 [file ijerph-15-01501-s001.pdf]

Genome-guided characterization of *Ochrobactrum* sp. POC9 enhancing sewage sludge utilization – biotechnological potential and biosafety considerations

Krzysztof Poszytek [1], Joanna Karczewska-Golec [1], Anna Ciok [2], Przemyslaw Decewicz [2], Mikolaj Dziurzynski [2], Adrian Gorecki [2], Grazyna Jakusz [1], Tomasz Krucon [1], Pola Lomza [1], Krzysztof Romaniuk [2], Michal Styczynski [2], Zhendong Yang [1], Lukasz Drewniak [1] and Lukasz Dziewit [2,*]

[1] Laboratory of Environmental Pollution Analysis, Faculty of Biology, University of Warsaw, Miecznikowa 1, 02-096 Warsaw, Poland; kposzytek@biol.uw.edu.pl, karczewska@biol.uw.edu.pl, grazyna.jakusz@biol.uw.edu.pl, tkrucon@biol.uw.edu.pl, pola.lomza@biol.uw.edu.pl, zyang@biol.uw.edu.pl, ldrewniak@biol.uw.edu.pl

[2] Department of Bacterial Genetics, Institute of Microbiology, Faculty of Biology, University of Warsaw, Warsaw, Poland; aciok@biol.uw.edu.pl, decewicz@biol.uw.edu.pl, mikolaj.dziurzynski@biol.uw.edu.pl, agorecki@biol.uw.edu.pl, romaniuk@biol.uw.edu.pl, mstyczynski@biol.uw.edu.pl, ldziewit@biol.uw.edu.pl

* Correspondence: ldziewit@biol.uw.edu.pl; Tel.: +48-225-541-406

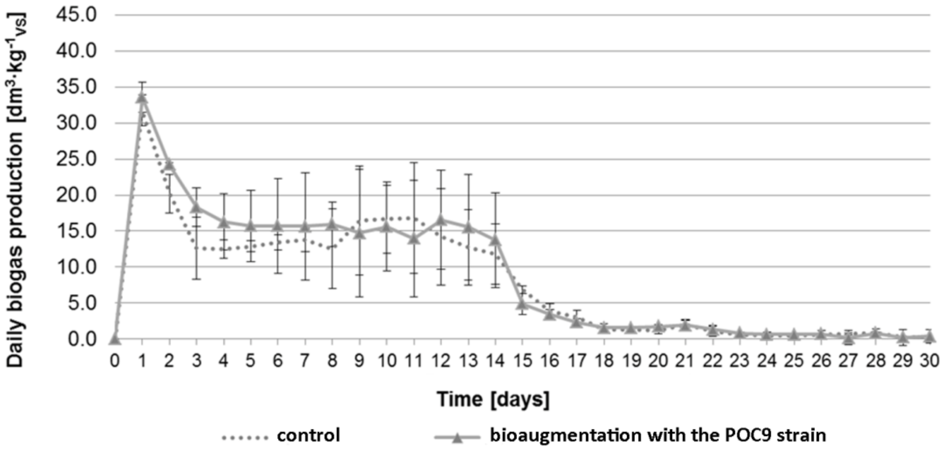

Figure S1. Daily biogas production during anaerobic digestion of sewage sludge.

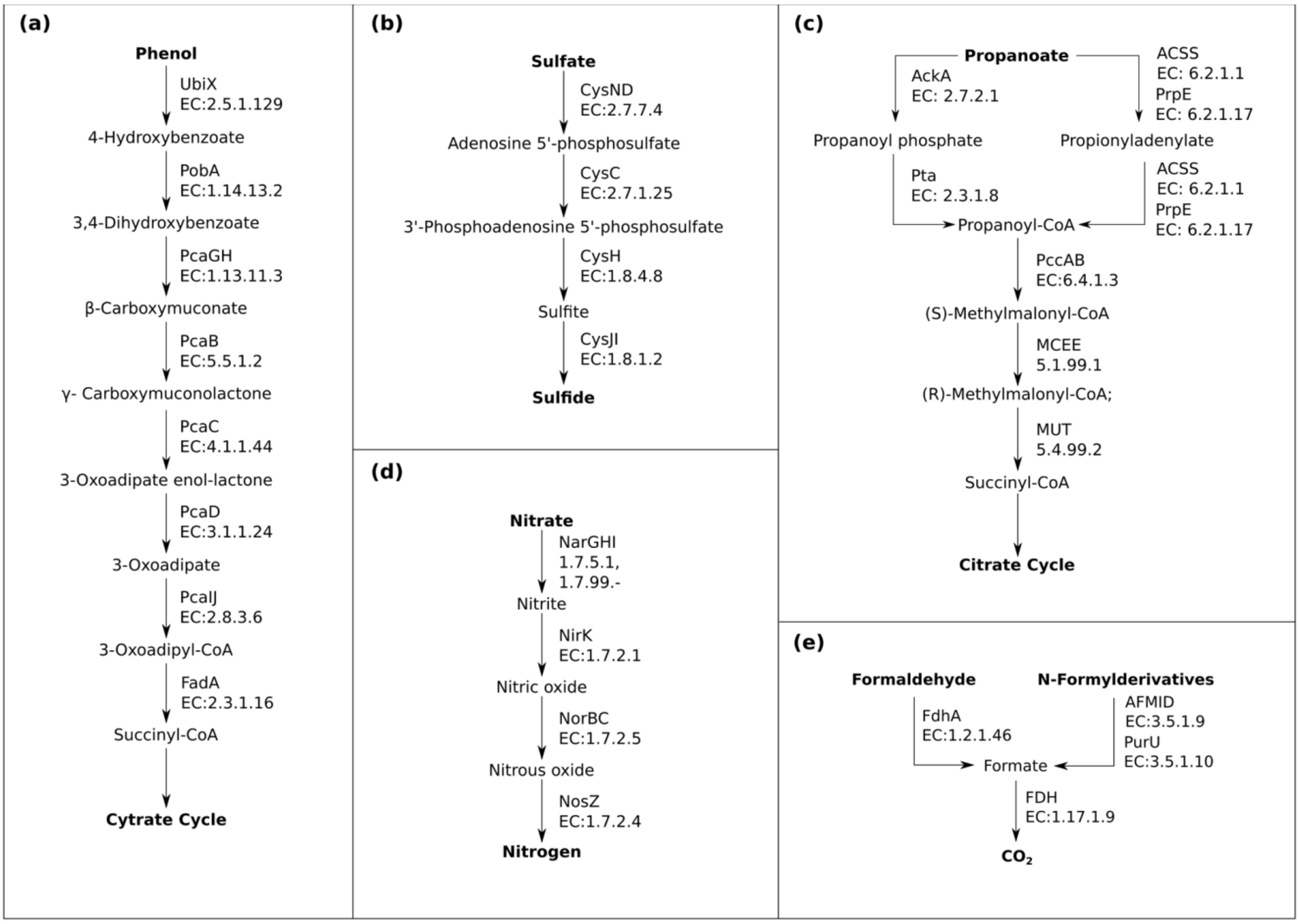

Figure S2. Metabolic pathways of a putative biotechnological value recognized within the POC9 draft genome. (a) Phenol degradation through benzoate utilization pathway. (b) Assimilatory sulfate reduction pathway. (c) Propanoate utilization pathway. (d) Denitrification pathway. (e) Decarboxylation of formate, formaldehyde and N-formyl derivatives pathway. The following abbreviations for enzyme names were used: AckA – acetate kinase, AFMID – arylformamidase, ACSS – acetyl-CoA synthetase, CysC – adenylyl-sulfate kinase, CysH – phosphoadenosine phosphosulfate reductase, CysJI – sulfite reductase (NADPH), CysND – sulfate adenylyltransferase, FadA – acetyl-CoA acyltransferase, FDH – formate dehydrogenase, FdhA – glutathione-independent formaldehyde dehydrogenase, MCEE – methylmalonyl-CoA/ethylmalonyl-CoA epimerase, MUT – methylmalonyl-CoA mutase, NarGHI – nitrate reductase, NirK – nitrite reductase, NorBC – nitric oxide reductase, NosZ – nitrous oxide reductase, PcaB – 3-carboxy-cis,cis-muconatecycloisomerase, PcaC – 4 carboxymuconolactone decarboxylase, PcaD – 3-oxoadipate enol-lactonase, PcaGH – protocatechuate 3,4-dioxygenase, PcaIJ – 3-oxoadipate CoA-transferase, PccAB – propionyl-CoA carboxylase, PobA – p-hydroxybenzoate 3-monooxygenase, PrpE – propionyl-CoA synthetase, Pta – phosphate acetyltransferase, PurU – formyltetrahydrofolate deformylase, UbiX – flavin prenyltransferase. Enzyme commission (EC) numbers for particular enzymes are presented.

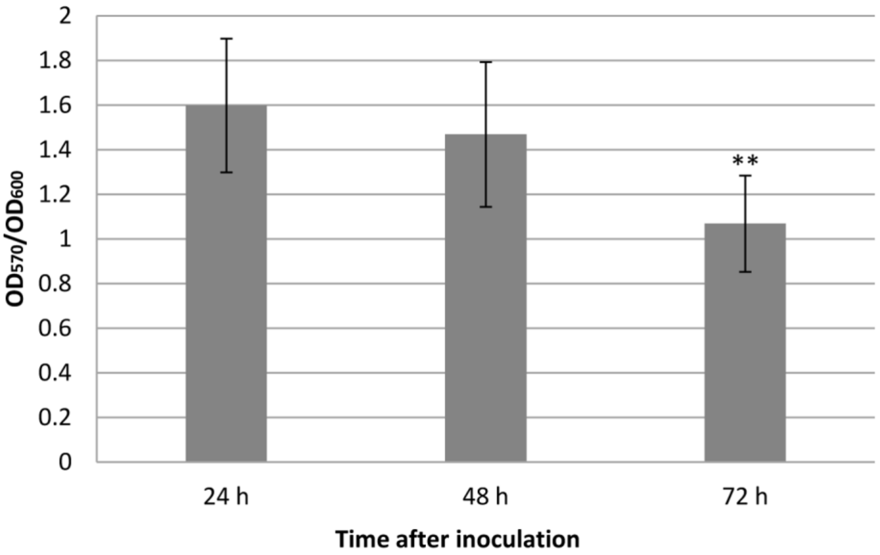

FIGURE S3. Adherence of *Ochrobactrum* sp. POC9 to an artificial (plastic) surface after three time intervals (24 h, 48 h and 72 h). ** – mean statistical significance $p<0.005$, compared to adherence after 24 h of incubaction.
